# Iron homeostasis and health: understanding its role beyond blood health – a narrative review

**DOI:** 10.1097/MS9.0000000000003100

**Published:** 2025-05-21

**Authors:** Emmanuel Ifeanyi Obeagu

**Affiliations:** Department of Biomedical and Laboratory Science, Africa University, Mutare, Zimbabwe

**Keywords:** anemia, iron homeostasis, metabolic health, nutritional balance, well-being

## Abstract

Iron is an essential trace element that plays a critical role in numerous physiological processes, including oxygen transport, cellular metabolism, immune function, and organ health. While its most well-known function is in hemoglobin synthesis for blood health, iron’s regulatory mechanisms extend far beyond the bloodstream. This review examines the broader implications of iron homeostasis, focusing on its impact on cellular functions, immune responses, and the health of organs such as the liver, heart, and brain. Disruptions in iron regulation, including both deficiency and overload, can lead to various disorders, including anemia, iron overload diseases, and chronic inflammation. Iron homeostasis is maintained by a complex balance of absorption, storage, and recycling, primarily regulated by hepcidin, a liver-derived hormone. Inadequate iron levels can impair mitochondrial function, DNA synthesis, and immune cell activation, leading to fatigue, anemia, and a weakened immune system. Conversely, excess iron can promote oxidative stress, contributing to tissue damage and conditions like hemochromatosis, cardiomyopathy, and neurodegenerative diseases. The liver, kidneys, heart, and brain are particularly sensitive to changes in iron balance, which can exacerbate or precipitate various health complications.

## Introduction

Iron is an essential element in human health, serving as a crucial cofactor in a wide array of biochemical processes. Beyond its well-established role in blood health through hemoglobin production and oxygen transport, iron is integral to cellular energy production, DNA synthesis, immune function, and the regulation of oxidative stress. The body maintains tight control over iron levels, ensuring its availability for critical physiological processes while preventing toxicity from iron overload. However, disturbances in iron homeostasis, such as deficiency or excess, can have profound effects on health^[1]^. Iron deficiency is one of the most common nutritional deficiencies worldwide, affecting approximately 1.62 billion people globally, with the highest prevalence among pregnant women and young children. According to the World Health Organization (WHO), iron deficiency anemia (IDA) affects 29.9% of the global population, representing a significant public health concern. Iron deficiency impairs numerous physiological functions, including mitochondrial energy production, immune response, and cognitive function. It is particularly detrimental during periods of rapid growth, such as childhood and pregnancy, and can result in long-term developmental and health consequences if left untreated^[2,3]^. While iron deficiency remains a significant health burden, iron overload disorders also pose substantial risks to human health. Hemochromatosis, a genetic condition characterized by excessive iron absorption, affects an estimated 1 in 200 to 1 in 300 individuals of European descent. Left untreated, hemochromatosis can lead to iron accumulation in organs such as the liver, heart, and pancreas, resulting in tissue damage, cirrhosis, diabetes, heart failure, and even cancer. Despite being less common than iron deficiency, iron overload disorders have serious long-term health consequences and are often undiagnosed until advanced stages^[4,5]^.

**HIGHLIGHTS**

- Iron regulation: Iron homeostasis is crucial for cellular metabolism, immune function, and organ health, beyond its role in blood health.
- Immune function: Iron plays a key role in modulating immune responses, influencing susceptibility to infections and inflammatory diseases.
- Organ health: Disrupted iron metabolism can lead to organ dysfunction, particularly in the liver, heart, and brain.
- Iron deficiency: Iron deficiency impairs overall health, contributing to fatigue, cognitive decline, and weakened immune defense.
- Therapeutic implications: Targeting iron homeostasis offers novel therapeutic strategies for managing iron-related disorders, including anemia and hemochromatosis.

Beyond blood health, iron plays a critical role in cellular metabolism. It is a central component of mitochondrial enzymes involved in energy production, particularly in the electron transport chain (ETC), where iron-containing proteins facilitate ATP synthesis. Iron is also essential for the function of ribonucleotide reductase (RNR), an enzyme required for DNA synthesis and cell division. As a result, iron is crucial for tissue growth and repair, and its deficiency can lead to impaired cellular proliferation, decreased energy production, and reduced tissue regeneration^[6]^. Iron also significantly impacts the immune system. Iron deficiency weakens the immune response, increasing susceptibility to infections, while iron overload can lead to chronic inflammation and contribute to the development of autoimmune diseases. The immune system utilizes iron in several ways, such as during the production of reactive oxygen species (ROS) by macrophages and neutrophils to combat pathogens. Additionally, iron is involved in the regulation of immune cell function, including the differentiation and activation of T lymphocytes, which are crucial for adaptive immunity^[7,8]^. Iron’s impact on organ function extends far beyond the blood and immune system. In the liver, iron is stored and released based on the body’s needs, and dysfunction in this process can lead to iron accumulation, causing liver damage, cirrhosis, and fibrosis. In the heart, iron deficiency leads to anemia, causing reduced oxygen delivery to tissues, while iron overload can result in cardiomyopathy and arrhythmias. The brain, too, is highly sensitive to iron imbalances, with iron deficiency being associated with cognitive impairments and developmental delays, while excess iron has been implicated in neurodegenerative diseases such as Alzheimer’s and Parkinson’s^[9]^.

Despite the critical importance of maintaining iron balance, managing iron-related disorders remains challenging. For instance, iron deficiency is often treated with oral iron supplements, but their efficacy can be limited by issues such as poor absorption and gastrointestinal side effects. On the other hand, managing iron overload typically involves phlebotomy or iron chelation therapy, but these treatments can be invasive or carry risks, such as iron deficiency if not carefully monitored. Furthermore, there is growing recognition of the need for more individualized treatments that target the underlying mechanisms of iron regulation, such as modulating hepcidin levels or targeting specific iron-dependent enzymes^[10]^. The global prevalence of IDA, particularly in low- and middle-income countries, calls for innovative strategies to address this widespread issue. Pregnant women and young children are particularly vulnerable, with IDA being a leading cause of maternal morbidity and mortality worldwide. According to the WHO, approximately 40% of pregnant women are affected by IDA, which can lead to adverse pregnancy outcomes, including preterm birth, low birth weight, and developmental delays in children. Improving iron nutrition and access to iron supplements is essential for addressing these public health challenges^[11]^. In contrast, iron overload disorders, though less common, are becoming increasingly recognized as a major cause of chronic disease. Hemochromatosis and other forms of iron overload, such as those seen in thalassemia or sickle cell disease, can lead to significant morbidity and mortality if left untreated. Early diagnosis and management are crucial to prevent organ damage and improve patient outcomes. Genetic screening and biomarkers for iron overload are advancing, allowing for earlier detection and better treatment options^[12]^. Iron’s role in human health is both broad and complex, and disruptions to iron homeostasis can have wide-ranging effects on well-being. While iron deficiency is more commonly recognized and treated, iron overload disorders are becoming increasingly acknowledged as critical health concerns. A better understanding of iron’s physiological functions beyond blood health, including its effects on cellular metabolism, immune function, and organ health, is essential for advancing both diagnostic and therapeutic strategies. Addressing the challenges of maintaining optimal iron levels and developing targeted interventions will be crucial for improving health outcomes on a global scale^[12]^.

## Aim

The aim of this review is to provide a comprehensive understanding of iron homeostasis beyond its traditional focus on blood health, highlighting its broader implications for cellular metabolism, immune function, and organ health.

## Review methods

### Search strategy

The review was conducted through a comprehensive literature search across multiple academic databases, including PubMed, Scopus, Google Scholar, and Web of Science. The search was performed using a combination of relevant keywords and medical subject headings terms such as “iron homeostasis,” “iron metabolism,” “iron deficiency,” “iron overload,” “cellular metabolism,” “immune system,” “iron and organ function,” and “iron-related disorders.” The search was conducted for articles published between 2000 and 2024 to ensure the inclusion of recent research developments.

### Inclusion and exclusion criteria

- Inclusion criteria:
  - Original research articles, clinical trials, and review papers discussing iron homeostasis and its role in various physiological systems, including cellular metabolism, immune response, and organ function.
  - Studies addressing the therapeutic implications of iron imbalances, including iron deficiency, overload, and modulation in disease contexts.
  - Articles that provided experimental evidence, clinical data, or insights into the mechanisms regulating iron metabolism and therapeutic strategies targeting iron dysregulation.

- Exclusion criteria:
  - Studies not directly related to iron metabolism, such as those focusing solely on unrelated aspects of cellular physiology.
  - Articles published before 2000, as the review aimed to focus on contemporary developments in iron homeostasis and therapeutic interventions.
  - Non-peer-reviewed literature, editorials, or opinion pieces without substantial scientific data.

### The physiology of iron homeostasis

Iron homeostasis refers to the tightly regulated balance between iron absorption, utilization, storage, and recycling within the body. Iron is a vital micronutrient, crucial for numerous physiological processes, including oxygen transport, DNA synthesis, and cellular energy production. However, both iron deficiency and overload can have severe consequences for health, making the regulation of iron homeostasis an essential aspect of maintaining overall well-being. The body has evolved complex mechanisms to control iron levels, ensuring that the amount of iron available is sufficient to meet metabolic needs without accumulating to toxic levels. Iron absorption primarily occurs in the small intestine, specifically in the duodenum and proximal jejunum. Iron enters the body mainly through dietary sources, which are divided into two forms: heme iron (from animal-based foods) and non-heme iron (from plant-based foods). Heme iron is absorbed more efficiently than non-heme iron. Once ingested, non-heme iron is reduced to its ferrous (Fe^2^⁺) form by the enzyme duodenal cytochrome B (Dcytb) to facilitate its absorption through the enterocyte (intestinal cell). Heme iron is absorbed intact by a specific heme transporter, HRG1. Once inside the enterocyte, iron binds to ferritin, a protein that serves as an intracellular iron storage molecule. The process is finely regulated by the hormone hepcidin, produced by the liver. Hepcidin binds to and degrades the iron exporter ferroportin, which is responsible for transporting iron from the enterocyte into the bloodstream. Hepcidin levels rise in response to high iron stores or inflammation, reducing iron absorption and preventing iron overload. Conversely, when iron stores are low, hepcidin levels decrease, allowing for increased iron absorption^[2,13,14]^.

### Transport and storage of iron

After absorption, iron is transported via the bloodstream by transferrin, a glycoprotein that binds to iron and facilitates its delivery to various tissues, including the bone marrow for red blood cell production. Transferrin binds to transferrin receptors (TfRs) on the surface of cells, allowing the iron–transferrin complex to be internalized and utilized. Once inside the cell, iron is either incorporated into enzymes and hemoglobin or stored in the form of ferritin or hemosiderin for future use. Iron storage occurs mainly in the liver, bone marrow, and spleen. Ferritin, the primary storage protein, can hold up to 4500 iron atoms in its hollow structure. When the body’s iron demand exceeds its immediate availability, ferritin stores are utilized to release iron. This ensures that iron is available when needed for processes such as hemoglobin production, mitochondrial function, and cellular metabolism^[15]^.

### Recycling of iron

A key aspect of iron homeostasis is the recycling of iron, especially from senescent red blood cells. Red blood cells have a lifespan of about 120 days, after which they are removed from circulation by macrophages in the spleen and liver. The heme group from hemoglobin is broken down into biliverdin, and iron is released in the process. The recycled iron is then transported back into the bloodstream via ferroportin and delivered to the bone marrow by transferrin for the production of new red blood cells. This recycling mechanism helps maintain iron balance in the body and reduces the need for continuous dietary iron intake. It is estimated that approximately 20–25 mg of iron is lost from the body daily, primarily through the shedding of skin cells, menstrual blood, and other minor sources. However, the majority of the body’s iron is recycled, highlighting the efficiency of iron metabolism^[16]^.

### Regulation of iron homeostasis

The regulation of iron homeostasis is a complex process, orchestrated by the interplay of various molecules and hormones. The primary regulator of iron absorption is hepcidin, a peptide hormone produced by the liver in response to iron levels, infection, or inflammation. Hepcidin levels increase when iron stores are sufficient or in the presence of inflammation, reducing iron absorption by the enterocytes and iron release from macrophages. Conversely, low iron levels or increased erythropoiesis (red blood cell production) stimulate the suppression of hepcidin, allowing for enhanced iron absorption and mobilization from stores. In addition to hepcidin, other molecules, such as ferroportin, transferrin, and ferritin, contribute to the regulation of iron homeostasis. Ferroportin controls the export of iron from enterocytes and macrophages into the bloodstream, while transferrin ensures the safe transport of iron to tissues. Ferritin serves as the storage protein for iron, helping to maintain a balance between iron availability and protection from oxidative damage. The role of the iron-responsive element (IRE)-iron regulatory protein (IRP) system also plays a critical role in the regulation of iron homeostasis. The IRE-IRP system controls the translation of key iron-related proteins, including ferritin and TfRs, based on cellular iron levels. When iron levels are low, IRPs bind to IREs in the untranslated regions of mRNA, preventing the translation of ferritin and promoting the synthesis of TfRs. This ensures that cells prioritize iron uptake when needed^[17,18]^.

### Dysregulation of iron homeostasis

Disturbances in iron homeostasis can lead to various health conditions, such as iron deficiency and iron overload. IDA is a common result of insufficient iron intake or impaired absorption, leading to reduced hemoglobin synthesis and decreased oxygen delivery to tissues. Symptoms of IDA include fatigue, weakness, and pale skin. On the other hand, excessive iron accumulation can lead to iron overload disorders, such as hereditary hemochromatosis, where the body absorbs too much iron and deposits it in organs, causing tissue damage. Other disorders related to dysregulated iron homeostasis include anemia of chronic disease (ACD), in which the body’s iron utilization is impaired due to inflammation or infection, and sideroblastic anemia, where defective heme synthesis leads to iron accumulation in mitochondria. These conditions highlight the complexity of iron regulation and the importance of maintaining an optimal balance for overall health^[19]^.

### Iron and cellular metabolism

Iron plays an indispensable role in cellular metabolism by supporting several crucial biochemical processes, particularly in energy production, DNA synthesis, and cellular respiration. As a key component of various enzymes and co-factors, iron is central to maintaining cellular function and supporting the energetic needs of cells. The unique ability of iron to readily accept and donate electrons makes it a vital element for processes that involve redox reactions, including the synthesis of adenosine triphosphate (ATP), the primary energy carrier in cells. Furthermore, iron’s involvement in oxygen transport and its influence on cellular signaling pathways underscore its importance in maintaining cellular homeostasis.

### Role of iron in ATP synthesis

Iron is a critical component of the mitochondrial enzymes involved in oxidative phosphorylation, a central pathway in cellular metabolism that generates ATP. The ETC is located in the inner mitochondrial membrane, where iron-containing proteins such as cytochrome c and iron–sulfur clusters play essential roles in electron transfer. These enzymes facilitate the movement of electrons through the ETC, which creates a proton gradient across the mitochondrial membrane. This gradient is then used by ATP synthase to generate ATP. Without iron, the enzymes involved in the ETC would not function properly, leading to diminished ATP production and compromised cellular energy supply. Additionally, iron is a necessary cofactor for several enzymes involved in the citric acid cycle (Krebs cycle), another central metabolic pathway. In this cycle, enzymes such as aconitase and succinate dehydrogenase contain iron–sulfur clusters that are critical for catalyzing metabolic reactions. The citric acid cycle generates electron carriers (NADH and FADH_2_) that feed into the ETC, providing the necessary electrons to fuel ATP production. Iron’s role in these key metabolic pathways is vital for maintaining cellular energy homeostasis, and any disruption in iron metabolism can impair cellular energy production, leading to cellular dysfunction^[20]^.

### Iron in DNA synthesis and cell division

Iron also plays a pivotal role in DNA synthesis and cell division, both of which are essential for cellular growth and maintenance. The enzyme RNR, which catalyzes the reduction of ribonucleotides into deoxyribonucleotides (the building blocks of DNA), requires iron for its activity. RNR contains iron atoms at its active site, and the proper function of this enzyme is critical for the replication of DNA. A deficiency in iron can impair DNA synthesis, leading to disruptions in cell division and potentially resulting in cellular arrest or apoptosis. Moreover, iron is essential for the synthesis of purines and pyrimidines, the nitrogenous bases that form the structure of DNA and RNA. In addition to its direct involvement in DNA synthesis, iron is also necessary for the proper function of several enzymes in the cell cycle, including those involved in DNA repair. This underscores the importance of iron in maintaining genomic integrity and preventing mutations or cellular damage that could lead to diseases such as cancer^[21]^.

### Iron and oxygen transport

One of the most well-known functions of iron is its role in oxygen transport. Iron is the key component of hemoglobin, the protein in red blood cells responsible for carrying oxygen from the lungs to tissues and organs throughout the body. Hemoglobin is a tetrameric protein that contains iron in its heme groups, which bind oxygen molecules. The reversible binding of oxygen to iron allows for the efficient delivery of oxygen to tissues in need of energy production. In addition to hemoglobin, iron is also a key component of myoglobin, a protein found in muscle tissues that facilitates oxygen storage and release during periods of high metabolic activity. This is particularly important for muscle function during exercise, where oxygen demand increases. By regulating the availability of oxygen, iron helps optimize cellular respiration and energy production, making it essential for normal metabolic function^[22]^.

### Iron and ROS production

Iron’s ability to participate in redox reactions also makes it a contributor to the production of ROS in cells. ROS are highly reactive molecules that can damage cellular structures, including lipids, proteins, and DNA, leading to oxidative stress. While ROS are an essential part of normal cellular metabolism and play important roles in cell signaling and immune responses, excessive ROS production can result in cellular injury and contribute to various diseases, including neurodegenerative conditions, cancer, and cardiovascular diseases. Iron is involved in the generation of ROS through the Fenton reaction, where iron in its ferrous (Fe^2^⁺) state reacts with hydrogen peroxide (H_2_O_2_) to produce hydroxyl radicals (•OH). These radicals are highly reactive and can cause extensive cellular damage. However, the body has evolved several mechanisms to manage iron-induced oxidative stress, such as antioxidant systems involving enzymes like superoxide dismutase, catalase, and glutathione peroxidase, which mitigate ROS damage. Proper regulation of iron homeostasis is critical to prevent excessive ROS production and oxidative damage^[23]^.

### Iron and cellular signaling pathways

Iron is not only involved in fundamental metabolic processes but also plays a role in cellular signaling. Iron acts as a regulator in several signaling pathways, particularly those related to cellular growth and differentiation. For instance, iron influences the activity of hypoxia-inducible factors (HIFs), which are transcription factors that help cells respond to low oxygen levels (hypoxia). Under normal oxygen conditions, HIFs are degraded; however, when oxygen levels are low, HIFs accumulate and initiate the expression of genes that help the cell adapt to hypoxia, such as those involved in angiogenesis and erythropoiesis. Iron also impacts other signaling pathways, including those regulating inflammation and cell survival. For example, iron’s influence on the immune system is mediated by its ability to regulate cytokine production and modulate the activity of immune cells. Additionally, iron can interact with kinases and phosphatases, which are enzymes involved in the phosphorylation of proteins, thereby influencing cell growth, survival, and apoptosis^[24]^.

### Disruption of iron homeostasis and metabolic diseases

When iron homeostasis is disrupted, it can lead to various metabolic disorders, either through iron deficiency or iron overload. Iron deficiency impairs cellular energy production, DNA synthesis, and cellular growth, leading to conditions such as anemia, fatigue, and developmental delays. On the other hand, iron overload, which can occur in disorders such as hemochromatosis, results in the accumulation of iron in organs like the liver, heart, and pancreas, leading to tissue damage, inflammation, and increased risk for conditions like cirrhosis, heart failure, and diabetes. Additionally, disruptions in iron homeostasis can contribute to the development of metabolic diseases, including insulin resistance and obesity. Iron overload has been implicated in the pathogenesis of these conditions, where excess iron in tissues can lead to oxidative stress and inflammation, impairing insulin signaling and glucose metabolism. Furthermore, the abnormal regulation of iron in certain cancers, where cancer cells may alter their iron metabolism to support rapid growth and proliferation, can contribute to tumor progression and drug resistance^[25]^.

### Iron and cancer metabolism

Iron’s role in cellular metabolism extends to cancer, where it influences tumor progression and survival. Cancer cells often exhibit altered iron metabolism, allowing them to maintain sufficient iron levels for rapid cell division and survival under metabolic stress. For instance, increased expression of TfRs and decreased expression of ferritin in cancer cells enhance iron uptake and utilization. This provides the necessary iron for high levels of DNA synthesis, cellular respiration, and antioxidant defense. Iron also modulates the response of cancer cells to chemotherapy and radiation therapy, with some tumors exploiting iron metabolism to resist treatment. Targeting the unique iron requirements of cancer cells has emerged as a potential therapeutic strategy, with the development of iron chelators and inhibitors of iron-dependent enzymes showing promise as adjuvants to conventional cancer treatments^[26]^.

### Iron and the immune system

Iron plays a critical role in the functioning of the immune system, influencing both innate and adaptive immune responses. As a central cofactor in many enzymes and cellular processes, iron is essential for the proliferation, differentiation, and activation of immune cells. Its role in immune function is multifaceted, affecting processes such as the production of ROS, lymphocyte activation, and cytokine secretion. However, the regulation of iron within the immune system is a delicate balance, as both iron deficiency and iron overload can have profound effects on immune responses and disease susceptibility.

### Iron’s role in innate immunity

Iron’s involvement in the innate immune system is particularly important for the activation and function of macrophages, neutrophils, and dendritic cells. Macrophages, which are key players in the innate immune response, require iron for the production of ROS during the process of phagocytosis. Iron is a crucial component of the enzymes involved in generating these ROS, which are used to kill ingested pathogens. In addition, iron is essential for the optimal function of iron-containing enzymes in the production of cytokines, such as interleukin-1 and tumor necrosis factor-alpha, which are key mediators of the immune response. Neutrophils, which are the first line of defense against bacterial infections, also rely on iron for the production of ROS through the NADPH oxidase complex. These ROS are instrumental in killing pathogens during the process of respiratory burst. Furthermore, iron affects neutrophil migration and activation, both of which are crucial for effective immune defense. The availability of iron in immune cells is therefore tightly regulated, as iron deficiencies can impair immune responses and increase susceptibility to infections, while iron overload can lead to excessive inflammation and tissue damage^[27]^.

### Iron and adaptive immunity

The adaptive immune system, which includes T lymphocytes and B lymphocytes, also depends on iron for optimal function. Iron is necessary for the proliferation and differentiation of both T and B cells, which are key players in the adaptive immune response. T cell activation, in particular, requires the availability of iron, as iron-dependent enzymes are involved in the signaling pathways that drive T cell proliferation. Additionally, iron is required for the synthesis of key molecules such as DNA and RNA during lymphocyte proliferation. B cells, which are responsible for antibody production, also rely on iron for their differentiation and function. Iron is involved in the synthesis of nucleotides, which are essential for DNA replication during the expansion of B cell populations. Furthermore, iron is necessary for the activation of enzymes involved in the formation of antibodies. Iron’s role in supporting lymphocyte function makes it crucial for adaptive immune responses to infections, vaccination, and immune memory^[28]^.

### Iron’s effect on immune cell differentiation and function

The impact of iron on immune cell differentiation is especially important in the context of immune regulation. Iron influences the differentiation of T helper cells (Th cells), which are critical for coordinating immune responses. For example, iron availability has been shown to affect the differentiation of Th17 cells, which play an essential role in fighting infections, particularly those caused by fungi and extracellular bacteria. On the other hand, excessive iron can lead to the expansion of regulatory T cells, which are involved in suppressing immune responses and maintaining immune tolerance. This balance of immune cell differentiation has significant implications for autoimmune diseases, infections, and cancer. Moreover, iron affects the polarization of macrophages into different phenotypes. M1 macrophages, which are pro-inflammatory and play a crucial role in fighting infections, require sufficient iron for their activation. Conversely, M2 macrophages, which are involved in tissue repair and immune tolerance, may be induced by higher iron levels. The ability to regulate the balance between M1 and M2 macrophages is critical for maintaining immune homeostasis and preventing chronic inflammation or excessive immune suppression^[29]^.

### Iron and immune modulation in disease

The immune-modulatory effects of iron are particularly evident in the context of disease. Infections, especially those caused by intracellular pathogens such as bacteria, viruses, and parasites, can lead to altered iron metabolism. Pathogens often manipulate the host’s iron metabolism to gain access to the iron required for their own growth. In response, the host’s immune system attempts to sequester iron to limit pathogen access. This is achieved through the upregulation of hepcidin, an iron-regulatory hormone that inhibits iron absorption and traps iron in macrophages, making it less available to pathogens. In autoimmune diseases, iron dysregulation can exacerbate inflammation and tissue damage. For example, in conditions like rheumatoid arthritis and systemic lupus erythematosus, iron overload has been shown to contribute to the inflammatory processes. Conversely, iron deficiency in individuals with autoimmune diseases can impair immune responses and increase the risk of infections. Therefore, the regulation of iron within the immune system is crucial for maintaining an appropriate immune response to pathogens while avoiding excessive inflammation that can result in autoimmune disorders^[30]^.

### Iron deficiency and immunity

Iron deficiency is one of the most common nutritional deficiencies globally and can have significant consequences for immune function. Iron deficiency impairs the function of both innate and adaptive immune cells, leading to increased susceptibility to infections. In particular, iron deficiency has been shown to impair the function of neutrophils, macrophages, and T lymphocytes. The reduced ability of these cells to respond to pathogens results in an increased risk of infections, particularly in individuals with compromised immune systems, such as children, the elderly, and individuals with chronic diseases. Iron deficiency also disrupts the production of key cytokines involved in immune responses, including those required for the activation of helper T cells and the recruitment of immune cells to sites of infection. Moreover, individuals with IDA have been shown to exhibit reduced vaccine efficacy, further emphasizing the importance of iron in maintaining an effective immune response to pathogens. Thus, addressing iron deficiency is critical for optimizing immune function and reducing the risk of infections^[31]^.

### Iron overload and immune system dysfunction

On the other hand, iron overload can have detrimental effects on immune system function. Excess iron can lead to the generation of ROS, which promote oxidative stress and inflammation. This can impair the function of immune cells and contribute to chronic inflammation, which is a hallmark of many autoimmune diseases, such as rheumatoid arthritis and inflammatory bowel disease (IBD). Furthermore, iron overload has been associated with impaired immune responses to infections, as excess iron can promote the growth of pathogens and hinder the immune system’s ability to clear infections. Iron overload can also affect the function of antigen-presenting cells (APCs), which are essential for initiating adaptive immune responses. These cells, which include dendritic cells and macrophages, rely on iron for proper antigen processing and presentation. Excess iron can impair APC function, leading to suboptimal activation of T and B cells. Additionally, the dysregulation of iron metabolism in immune cells can contribute to the development of cancer by promoting immune evasion and tumor progression^[32]^.

### Iron in organ function

Iron is a vital micronutrient that plays a crucial role in the functioning of nearly every organ system in the body. Its involvement is particularly essential in processes like oxygen transport, cellular respiration, and metabolism. The homeostasis of iron within the body is tightly regulated to ensure it is available where needed while preventing toxic excess. The most well-known role of iron is its incorporation into hemoglobin, the protein responsible for oxygen transport in red blood cells, but its significance extends well beyond blood health. Iron is involved in various biochemical processes that support the health and function of vital organs, including the brain, liver, heart, and kidneys.

### Brain function and iron

Iron is indispensable for brain function and plays a pivotal role in maintaining cognitive health. Iron is involved in the synthesis of neurotransmitters such as dopamine, serotonin, and norepinephrine, which are crucial for mood regulation, attention, and learning. Iron also supports myelination, the process by which nerve cells are coated with a protective layer that enhances the speed of electrical signals. Deficiencies in iron can impair the function of the central nervous system (CNS), leading to cognitive and behavioral issues such as poor attention span, learning difficulties, and in severe cases, developmental delays. Iron deficiency during pregnancy and early childhood is particularly detrimental, potentially resulting in long-lasting neurodevelopmental issues. Inadequate iron levels in the brain can lead to fatigue, reduced cognitive function, and mood disturbances and have been linked to conditions such as attention deficit hyperactivity disorder (ADHD) and depression. Conversely, iron overload in the brain is associated with neurodegenerative diseases, including Alzheimer’s disease and Parkinson’s disease. Excess iron can catalyze the formation of free radicals through the Fenton reaction, leading to oxidative stress and damage to neurons. Research has shown that impaired iron regulation in the brain may contribute to the pathophysiology of these diseases, as abnormal iron accumulation in the basal ganglia and other regions of the CNS has been observed in these conditions. The relationship between iron dysregulation and neurodegenerative diseases is an area of ongoing research, with potential therapeutic implications for iron chelation in these disorders^[2,10–12]^.

### Liver function and iron

The liver plays a central role in maintaining iron homeostasis through the regulation of iron absorption, storage, and release. Hepatocytes in the liver store excess iron in the form of ferritin and regulate the release of iron into the bloodstream via the iron-regulatory hormone hepcidin. Hepcidin controls iron levels by inhibiting iron absorption from the intestine and limiting iron release from macrophages and liver stores. The liver is therefore essential for ensuring that iron levels are balanced, preventing iron deficiency or overload. Iron overload in the liver, particularly in conditions such as hereditary hemochromatosis, can lead to significant liver damage. Excess iron accumulation in the liver causes hepatocellular damage and increases the risk of developing cirrhosis, liver fibrosis, and liver cancer. This excess iron catalyzes the production of free radicals, which in turn promote oxidative stress, inflammation, and fibrosis. Furthermore, individuals with chronic liver diseases, including non-alcoholic fatty liver disease, may also experience iron accumulation in the liver, exacerbating liver injury and increasing the risk of progressive disease. Thus, the liver’s ability to maintain proper iron homeostasis is crucial for preventing liver dysfunction and associated complications^[13–15]^.

### Cardiovascular function and iron

Iron is vital for cardiovascular health due to its role in oxygen transport and cellular energy metabolism. Iron is a key component of hemoglobin, the protein in red blood cells responsible for carrying oxygen from the lungs to tissues and organs. This oxygen delivery is essential for maintaining the function of heart muscles and other tissues in the cardiovascular system. Anemia, which is often caused by iron deficiency, can result in reduced oxygen supply to tissues, leading to fatigue, weakness, and cardiovascular complications, particularly in individuals with preexisting heart conditions. Iron is also involved in the function of enzymes within the heart muscle and vascular endothelium. For instance, iron-containing enzymes like nitric oxide synthase are involved in the regulation of blood vessel dilation and blood pressure. Nitric oxide, produced by these enzymes, plays a critical role in maintaining vascular tone and ensuring adequate blood flow. Iron deficiency can impair nitric oxide production, contributing to endothelial dysfunction, which is a key factor in the development of cardiovascular diseases such as atherosclerosis and hypertension. On the other hand, iron overload has been associated with an increased risk of heart disease, as excess iron can lead to oxidative damage in the heart and blood vessels, contributing to heart failure and arrhythmias^[16–19]^.

### Kidney function and iron

The kidneys, like other organs, require iron for optimal function. Iron is involved in several renal processes, including the synthesis of erythropoietin (EPO), a hormone produced by the kidneys that regulates red blood cell production. EPO production is stimulated by hypoxia, and iron is essential for its synthesis, as it is required for the function of prolyl hydroxylase, the enzyme responsible for regulating the stability of the HIF, which in turn regulates EPO production. In iron deficiency, EPO production can be impaired, leading to anemia, a common complication in chronic kidney disease (CKD). Moreover, iron homeostasis in the kidneys is influenced by the levels of hepcidin, the hormone that regulates systemic iron metabolism. In CKD, elevated hepcidin levels contribute to iron retention in the kidneys, which can exacerbate the disease by promoting inflammation and fibrosis. The accumulation of iron in renal cells can lead to oxidative stress, nephron damage, and eventually kidney dysfunction. Iron supplementation in patients with CKD must therefore be carefully managed, as both iron deficiency and iron overload can negatively impact kidney function^[20,21]^.

### Iron and endocrine function

Iron also plays a role in the endocrine system, influencing the function of various hormones. For example, iron is involved in the synthesis of thyroid hormones, which are critical for metabolism, growth, and development. Iron deficiency can impair thyroid hormone production, leading to hypothyroidism and its associated symptoms such as fatigue, weight gain, and depression. Additionally, iron affects insulin sensitivity and glucose metabolism. Studies have shown that iron deficiency is linked to increased insulin resistance, which can exacerbate conditions like type 2 diabetes. In contrast, iron overload can disrupt endocrine function through its effects on various glands, including the pituitary and pancreas. Excess iron accumulation can impair the secretion of hormones such as growth hormone and insulin, contributing to metabolic disorders. The interaction between iron and the endocrine system highlights the need for careful management of iron levels to maintain overall organ health^[22]^.

### Iron in muscle function

Iron also plays a critical role in muscle function, particularly in the processes of oxygen transport and energy production. Iron is an essential component of myoglobin, a protein found in muscle cells that stores and transports oxygen. During physical activity, muscles require increased oxygen for energy production, and myoglobin helps facilitate this process. Iron deficiency can impair myoglobin function, leading to reduced oxygen availability in muscles, resulting in fatigue, decreased exercise performance, and muscle weakness. Iron is also involved in cellular respiration within muscle cells, as it is a cofactor for several enzymes in the ETC, the pathway through which cells produce ATP, the energy currency of the body. Iron deficiency can reduce ATP production, limiting muscle performance and endurance. Iron supplementation has been shown to improve muscle function and exercise capacity, especially in individuals with IDA^[23]^.

### Disorders of iron metabolism

Iron metabolism is a finely regulated process that is essential for maintaining proper cellular function and overall health. Disorders of iron metabolism occur when this regulation is disrupted, leading to either an excess or deficiency of iron in the body. Both iron deficiency and iron overload have significant implications for various organ systems, causing a wide range of health issues. These disorders can be classified into two main categories: iron deficiency disorders and iron overload disorders, each with distinct pathophysiological mechanisms and clinical manifestations.

### Iron deficiency disorders

Iron deficiency is the most common nutritional deficiency worldwide and can lead to IDA, the most prevalent form of anemia globally. Iron is essential for the production of hemoglobin, the protein in red blood cells responsible for carrying oxygen. Without adequate iron, the body is unable to produce sufficient hemoglobin, leading to a reduced ability to transport oxygen. IDA is characterized by fatigue, pallor, weakness, shortness of breath, dizziness, and impaired cognitive function, particularly in children and pregnant women. The most common causes of iron deficiency include inadequate dietary intake, increased iron requirements during pregnancy or growth, blood loss (e.g., gastrointestinal bleeding or heavy menstruation), and impaired iron absorption (e.g., due to celiac disease or gastric surgeries). Chronic iron deficiency can lead to more severe symptoms, including cognitive impairments, compromised immune function, and developmental delays in children. In severe cases, it can result in heart failure or even death due to the strain placed on the cardiovascular system from insufficient oxygen delivery to tissues. Treatment for iron deficiency typically involves iron supplementation and addressing the underlying cause of the deficiency. Oral iron supplements are commonly used, but in severe cases, intravenous iron or blood transfusions may be required. Dietary changes to increase iron intake and improve iron absorption, such as the consumption of heme iron (found in animal products) and the use of vitamin C to enhance absorption, are also recommended^[24,25]^.

### Iron overload disorders

Iron overload occurs when the body accumulates excess iron, which can be toxic to organs and tissues. Unlike iron deficiency, where iron levels are insufficient, iron overload disorders result from an inability to regulate iron absorption, storage, and release properly. The most common disorder associated with iron overload is hereditary hemochromatosis. This genetic condition results from mutations in the HFE gene, leading to excessive absorption of dietary iron in the intestines and subsequent deposition of iron in various organs, particularly the liver, heart, and pancreas. Over time, this excess iron causes damage to these organs, leading to conditions such as cirrhosis, heart failure, diabetes, and arthritis. Other causes of iron overload include transfusion-related iron overload, which occurs in patients receiving frequent blood transfusions, such as those with thalassemia or sickle cell anemia. Repeated transfusions introduce large amounts of iron into the body, which the body cannot excrete efficiently, leading to iron accumulation in tissues. The excess iron can damage organs, particularly the liver and heart, and lead to complications like cardiac arrhythmias, liver fibrosis, and endocrine dysfunction. Excessive iron can also result from other rare conditions, such as ferroportin disease and TfR 2 mutations, which disrupt normal iron transport and storage. In these conditions, iron builds up in the body despite normal or slightly increased hepcidin levels, a hormone that normally reduces iron absorption. The management of iron overload disorders involves the use of iron chelation therapy, which binds to excess iron and promotes its excretion through urine or feces. Regular phlebotomy (blood removal) is also an effective treatment for hereditary hemochromatosis, as it reduces iron stores by encouraging the body to produce new blood cells. However, treatment must be tailored to the underlying cause of the disorder to prevent organ damage^[26–28]^.

### Other iron metabolism disorders

In addition to iron deficiency and overload disorders, there are several other rare conditions that impact iron metabolism. ACD, also known as anemia of inflammation, is a type of anemia that occurs in response to chronic infection, inflammation, or malignancy. In ACD, iron is sequestered in macrophages and other storage sites due to increased hepcidin levels, resulting in a functional iron deficiency despite normal or elevated total body iron stores. This condition is commonly seen in patients with conditions such as rheumatoid arthritis, CKD, and certain cancers. Iron-refractory IDA (IRIDA) is a rare inherited disorder that results in severe iron deficiency that does not respond to oral iron supplementation. It is caused by mutations in the TMPRSS6 gene, which leads to impaired regulation of hepcidin, a key regulator of iron homeostasis. Patients with IRIDA require more aggressive treatment, including intravenous iron or red blood cell transfusions. Sideroblastic anemia is a group of inherited or acquired disorders characterized by defective heme synthesis in red blood cell precursors, leading to the accumulation of iron in the mitochondria of developing red blood cells. This disorder can result in ineffective erythropoiesis, iron overload, and anemia. The underlying genetic mutations vary, but they generally affect enzymes involved in heme production, such as ALAS2. Finally, mucosal immune iron deficiency can occur in individuals with gastrointestinal diseases, such as celiac disease or IBD, where inflammation in the gut impairs iron absorption. This form of iron deficiency is often overlooked due to its gradual onset and subtle clinical symptoms but can be identified through laboratory tests showing low iron levels, low transferrin saturation, and elevated ferritin levels, indicative of underlying inflammation^[29,30]^.

### Therapeutic implications of iron homeostasis

Iron homeostasis plays a central role in maintaining physiological balance and is critical for proper organ function, cellular metabolism, immune response, and overall health. The therapeutic implications of iron homeostasis extend far beyond the treatment of IDA or iron overload disorders, highlighting its importance in various diseases and conditions. By understanding and manipulating iron metabolism, it is possible to develop novel therapeutic strategies that target both deficiency and excess iron, thereby improving patient outcomes across a wide spectrum of medical conditions.

### Targeting iron deficiency: restoration of iron balance

Iron deficiency remains one of the most common nutritional disorders worldwide, leading to anemia, fatigue, cognitive impairments, and increased susceptibility to infections. Effective management of iron deficiency involves replenishing iron stores and ensuring adequate iron availability for the production of hemoglobin and other iron-dependent enzymes. Traditional iron supplementation through oral or intravenous iron remains the cornerstone of treatment for IDA, but emerging therapeutic strategies are increasingly being explored to improve outcomes for patients with chronic or severe deficiency. Recent advancements in iron therapy include nanoparticle-based iron formulations and liposomal iron delivery systems, which offer improved bioavailability and reduced gastrointestinal side effects compared to conventional iron salts. These innovative formulations are designed to enhance iron absorption, minimize oxidative stress, and improve patient compliance, particularly in individuals with chronic conditions requiring long-term iron supplementation. In some cases, such as during pregnancy or in patients with IBDs or malabsorption syndromes, intravenous iron therapy may be necessary for effective treatment, especially when oral iron fails to meet the body’s needs. Moreover, erythropoiesis-stimulating agents (ESAs) are being used in conjunction with iron therapy to promote red blood cell production in patients with anemia associated with CKD, cancer, or inflammatory conditions. The combination of iron supplementation with ESAs can help optimize treatment outcomes by addressing both the iron deficiency and the underlying anemia. However, careful monitoring of iron status is essential to prevent iron overload, which can occur with excessive iron supplementation in these patients^[28,29]^.

### Iron overload

On the other side of the spectrum, iron overload represents a major clinical challenge, with conditions like hereditary hemochromatosis, transfusion-dependent anemia, and other disorders resulting in excessive iron accumulation. Accumulation of iron in vital organs such as the liver, heart, and pancreas can lead to organ damage, including cirrhosis, heart failure, diabetes, and arthritis. Therefore, therapeutic approaches in managing iron overload aim to reduce the burden of excess iron while minimizing its toxic effects. The primary treatment strategy for iron overload is iron chelation therapy, which involves the use of agents that bind to excess iron and facilitate its excretion from the body. Deferoxamine, deferasirox, and deferiprone are the most widely used iron chelators for managing transfusion-related iron overload. These chelators help reduce iron stores by binding to iron and promoting its elimination through the urine or feces. However, the effectiveness of iron chelation depends on early diagnosis, regular monitoring of iron levels, and adherence to long-term treatment regimens, as iron accumulation can be insidious and irreversible if left untreated. Iron chelation therapy is particularly crucial for patients with thalassemia or sickle cell anemia who require frequent blood transfusions. While blood transfusions provide life-saving treatment, they also introduce significant amounts of iron into the body. Over time, this excess iron can cause substantial organ damage if not managed through appropriate chelation therapy. In some cases, organ transplantation or other interventions may be required in patients with severe iron overload. In addition to chelation therapy, phlebotomy (therapeutic blood donation) is another effective treatment option for hereditary hemochromatosis and some forms of transfusion-related iron overload. Regular blood removal can reduce iron stores and prevent organ damage. However, this approach is not suitable for all patients, particularly those with conditions such as anemia or low blood counts^[30,31]^.

### Modulating iron metabolism for disease treatment

Iron metabolism not only is crucial for the treatment of iron deficiency and overload but also plays an important role in the pathogenesis of various diseases, including cancer, neurodegenerative disorders, and cardiovascular diseases. In cancer, the role of iron is complex, as iron is necessary for tumor growth but can also contribute to oxidative stress and cell death. Tumor cells often have altered iron metabolism to meet their increased demand for iron, leading to aberrant expression of iron-regulatory proteins such as TfR 1 (TfR1), ferroportin, and hepcidin. Targeting these pathways presents a promising therapeutic strategy for cancer treatment. Iron chelation in cancer therapy is gaining traction as a potential strategy to deprive tumor cells of iron and inhibit their growth. By limiting the availability of iron, iron chelators can suppress tumor progression and metastasis. Several studies are investigating the efficacy of iron chelation in combination with other therapies, such as chemotherapy or radiation therapy, to enhance treatment outcomes. However, clinical trials are still ongoing to establish the safety, optimal dosages, and effectiveness of iron chelation in cancer therapy. In neurodegenerative diseases such as Alzheimer’s and Parkinson’s, iron accumulation in the brain has been implicated in neuroinflammation, oxidative stress, and neuronal damage. The development of drugs that can regulate iron homeostasis in the brain, potentially by targeting brain-specific iron transporters or modulating iron absorption in the CNS, is an area of growing interest in neurotherapeutics. Iron modulation may offer a new avenue for the treatment of these debilitating disorders. Furthermore, in cardiovascular diseases, particularly atherosclerosis, altered iron homeostasis has been linked to endothelial dysfunction and oxidative damage. Targeting iron metabolism may offer novel therapeutic opportunities for reducing oxidative stress, inflammation, and plaque formation in patients with cardiovascular diseases. However, the balance between iron deficiency and overload must be carefully managed to avoid exacerbating cardiovascular complications^[30–32]^.

## Conclusion

Iron homeostasis is fundamental to maintaining health and well-being, as it influences critical physiological processes such as cellular metabolism, immune function, and organ performance. Its regulation is essential not only for the prevention and management of iron deficiency but also for the avoidance of iron overload, both of which can lead to a wide range of health complications. The therapeutic implications of iron homeostasis are vast, spanning from the treatment of iron-deficiency anemia with novel formulations and delivery systems to the management of iron overload through chelation therapy and other interventions.

## Data Availability

Not applicable as this is a review.

## Research registration unique identifying number (UIN)

Not applicable as this is a review.
